# Veterinary Colleges—They Come

**Published:** 1892-11

**Authors:** 


### EDITORIAL.

---

### VETERINARY COLLEGES—THEY COME.

Well ! we Americans are a great people. What we have not, we endeavor to get, and all that we have obtained, good or bad, we are apt to hang on to for fear that some class of society might exist or develop and find that their peculiar faculties were not provided for. There are various sorts of veterinary schools in the American continent. There are schools which were established with the primary object of advancing the status of veterinary education, in which the labors of those interested have been given with whole-soul enthusiasm, having in view only the development of students who should advance their profession. There are schools organized and carried on for the purpose of making money. Some of them have succeeded and made money for their organizers, either directly by the numerous checks of a heterogeneous lot of neophytes who have been rushed through, with few cullings—some to succeed by their natural ability, and many to fail because they have no foundation to attempt a professional practice upon ; or, the founders have found success indirectly in the advertisement which these diploma mills have given them, before a superficial general public.

Some schools have hampered their financial success by enforcing three years of study, while others have helped their coffers by advertising but two short winters and accepting the check and registration of students as the equivalent for one year's study. Some have held public examinations, after which the unsuccessful candidate could blame no one but himself for his failure, while others employ teachers like their stable attendants to swell their catalogue pages, and diploma students who have proved themselves deficient in the first elements of their studies.

After a year's mature deliberation, the United States Veterinary Medical Association, at its last annual meeting, decided that its ranks should only in future be filled from graduates who have had at least a three-years' course of study in schools in which the teachers have fulfilled certain qualifications. At this same meeting one of the most prominent veterinarians of the United States was seriously considered for the presidency of the Association, on account both of his individuality and because of his public position. Within a few weeks, to the file of catalogues of the various veterinary institutions, ranging from fully organized schools to agricultural colleges, in which the total technical instruction is given by one veterinarian, is added a new one. We now have in the United States a "NATIONAL VETERINARY COLLEGE," with untold advantages. It is situated at Washington, D. C., the "capital of this great stock-growing country." "It is central and easily accessible from the North and the South, the East and the West." The first page of its catalogue advertises that the future "Vet." can buy his books in New York and the last pages inform him that he can buy his instruments in Chicago and that he can sell or exchange "law books, Government publications, theological books, and his old school books," for such literature as he needs in Washington. This will be a valuable assistance to the farmer's boy who is shortly to join the ranks of the profession. He can collect from the generous gifts of his member of Congress the reports of the various departments at Washington, the speeches of Mr. Hayseed on ruta baga, and of Mr. Tarif on the McKinley bill, and sell them for enough to pay his board. The law and theological students who have failed in their original vocations and think that the voiceless domestic animals will find them more harmonious than the two-legged searchers for advice, counsel and salvation, can exchange their copies of the apocrophy and records of the police courts for the "Live Stock Encyclopædia," and "Every Man His Own Horse Doctor," and for Uncle Jerry Rusk's book on the Horse.

Should, however, the embryo Vet. not be a clever dealer in old junk, he has an attractive offer of the libraries of Congress, the Surgeon-General and the Department of Agriculture at his disposal, and these with the museums at Washington would, at first thought, seem great advantages, but the next page of the catalogue indicates that the Government employees, who have founded this school are not good mathematicians, for they inform us that they give a *six* months' course, commencing October 18th and ending in March. We next recognize that the regular course has but two sessions, which experience has taught, does not leave much time for reading, and, the United States Veterinary Medical Association has decided, does not fit a veterinarian to become one of its members. Special features of the education promised are "designed to fit the students for the many responsible and lucrative positions which have been opened up to competent men, by recent State and National Legislation."

The dolicho-cephalic practitioner of the future will train his office students in pairs—one a Republican and one a Democrat—then the vicissitudes of politics will only lead to convenient holidays and refreshing exchange of work for the young men. The Democratic boy who has labored in the infirmary, mixing balls and blistering mules' heels, will look for a change of government to give him an arm chair at the Helminthological tables under the roof of the Bureau of Animal Industry, or for a roving commission to look for C. P. P. in Long Island and the races at Coney Island. The disappointed Republican boy may be happy to return to the domestic circle, but the preceptor can always be sure both of an assistant at home and of a government salary for his other protegé. Twenty-one years of age, a good moral character and about the usual fee of all the other colleges are part of the requisites for a diploma from this new school. The Roster of the faculty contains the names of veterinarians and other scientists who have been well-known for their intelligence and professional ability, and who are popular social companions We regret that they should have loaned themselves to the foundation of another two years school.

HUIDEKOPER.

